# A Genetically Informed Longitudinal Study of Loneliness and Dementia Risk in Older Adults

**DOI:** 10.3389/fgene.2021.661474

**Published:** 2021-09-17

**Authors:** Alice J. Kim, Alaina I. Gold, Laura Fenton, Matthew J. D. Pilgrim, Morgan Lynch, Cailin R. Climer, Eric N. Penichet, Alyssa Kam, Christopher R. Beam

**Affiliations:** ^1^Department of Psychology, University of Southern California, Los Angeles, CA, United States; ^2^School of Gerontology, University of Southern California, Los Angeles, CA, United States

**Keywords:** loneliness, dementia, aging, behavior genetics, longitudinal analysis

## Abstract

Although several studies have shown small longitudinal associations between baseline loneliness and subsequent dementia risk, studies rarely test whether change in loneliness predicts dementia risk. Furthermore, as both increase with advancing age, genetic and environmental selection processes may confound the putative causal association between loneliness and dementia risk. We used a sample of 2,476 individual twins from three longitudinal twin studies of aging in the Swedish Twin Registry to test the hypothesis that greater positive change in loneliness predicts greater dementia risk. We then used a sample of 1,632 pairs of twins to evaluate the hypothesis that effects of change in loneliness on dementia risk would remain after adjusting for effects of genetic and environmental variance. Phenotypic model results suggest that mild levels of baseline loneliness predict greater dementia risk. Contrary to our hypothesis, change in loneliness did not correlate with dementia risk, regardless of whether genetic and environmental selection confounds were taken into account. Worsening loneliness with age may not confer greater dementia risk.

## 1. Introduction

Study of the association between perceived social isolation (i.e., loneliness) and dementia risk has intensified over the last 10 years. Three systematic reviews have been published in the last 3 years, one of which demonstrated a null association between loneliness and dementia risk (Penninkilampi et al., 2018) and two of which reported the same small mean effect (Cohen's *d* = 0.13) on similar sets of studies (Lara et al., 2019; Solmi et al., 2020). Increased interest in the association between loneliness and dementia risk has raised questions about the underlying processes that transform loneliness into observable symptoms of dementia. In the current report, we investigate whether greater increases in loneliness increases dementia risk and whether their association is more consistent with social selection hypotheses or social causation hypotheses.

Loneliness and dementia are phenomena of increased age, leading to the general hypothesis that age-related increases in loneliness increase dementia risk. The greatest known risk factor of dementia is age (Hebert et al., 2013; Beam et al., 2018), and loneliness levels increase, on average, from young-old age through old-old age (Beam and Kim, 2020). One potential theory that could explain late-life increases in loneliness is socioemotional selectivity theory, a lifespan theory that explains how motivations for interpersonal investment change with age (Carstensen, 1992). During young and middle adulthood, people's motivations for social connection change, resulting in a shift from maintenance of many weak social ties to selective investment in a few strong social ties. Such social selectivity, however, is not free of negative consequences. As close intimates pass away with age, they cannot be replaced easily, possibly putting older adults at increased risk for loneliness across older adulthood. Investment in fewer interpersonal relationships might be an adaptive strategy for minimizing loneliness during people's middle years but may become an inefficient, and potentially maladaptive, interpersonal strategy in old age, particularly in the same period of the lifespan in which dementia risk also increases. Age-related increases in loneliness, thus, would be expected to correlate with dementia risk. No study to date, however, has examined whether individual differences in intraindividual change in loneliness predicted dementia risk. Although previous studies have focused on linear growth in loneliness (Wilson et al., 2007), which may misfit natural change in loneliness, we examine whether differences in non-linear trajectories of loneliness predict dementia risk.

In addition to the question of whether change in loneliness predicts dementia risk, a problem not often dealt with in the literature is whether effects of loneliness on dementia risk reflect a causal association or selection processes. Findings implicitly assume that greater levels of loneliness increases risk of dementia (Sundström et al., 2020), although this may not be a true effect. As random assignment to loneliness and dementia is unethical and impractical, one of the primary difficulties in the literature has been disentangling whether the association between loneliness and dementia risk results from social causation, social selection, or both. Under the *social causation hypothesis*, loneliness itself increases risk of dementia, whereas under the *social selection hypothesis*, dementia risk among lonelier people is more likely to occur because of their greater underlying biological and environmental risk exposure. As the majority of findings indicate that associations between loneliness and dementia risk are attenuated after adjusting for covariates (e.g., depressive symptomatology, see Holwerda et al., 2014), putative causal effects of loneliness on dementia risk are uncertain. Thus, it is important to use research designs that can clarify direct causal effects, if any, of loneliness on dementia risk. Genetically informed research designs, such as twin studies, are one solution to infer causation as they adjust for unmeasured genetic and environmental selection confounds that may obscure causal associations (Turkheimer and Harden, 2014; Beam and Turkheimer, 2017). Selection processes, like a genetic predisposition for longevity, may account for their correlation. As such, we also evaluate whether effects of individual growth parameters of loneliness on dementia risk remain statistically significant after adjusting for genetic and environmental selection that might confound their association.

Twin designs are an efficient way to test the potentially causal role that loneliness has for dementia risk. Genetic and environmental factors shared by twins cannot explain why twins with greater positive rates of change in loneliness might also be more likely to be diagnosed with dementia. In pairs of monozygotic twins, for example, differences in the effect of loneliness on dementia risk must be attributed to twins' differences in environmental exposure, as they share all of their genotype and common environments. These effects are regarded as quasi-causal in the sense that twin studies lack random assignment and so are vulnerable to third variable confounds like all observational studies.

In the current study, we use a sample of 2,476 individual twins from three longitudinal twin studies of aging to test whether age-related increases in loneliness predict dementia risk. We further test whether these effects are consistent with social causation hypotheses, social selection hypotheses, or a combination of both. To test this, we decompose the variances in loneliness growth parameters into genetic and shared environmental sources of variance to test whether genetic, environmental, or both sources accounted for effects of loneliness on dementia risk. As the ε4 allele of the APOE gene is the single causal variant associated with late-onset Alzheimer's disease risk and nearly 75% of all dementia cases are Alzheimer's disease cases (Beam et al., 2018), APOE ε4 allele status also was included in the current study. In this way, both measured and unmeasured genetic influences underlying dementia risk were included in the current study.

## 2. Materials and Methods

### 2.1. Participants

The study sample was drawn from three longitudinal studies of aging in the Swedish Twin Registry: the Swedish Adoption/Twin Study of Aging (SATSA; Finkel and Pedersen, 2004), Aging in Men and Women study (GENDER; Gold et al., 2002), and Origins of Variance in the Oldest Old: Octogenarian Twins (OCTO-Twin; McClearn et al., 1997). SATSA began in November 1984 and ended January 2015, GENDER began in November 1994 and ended July 2007, and OCTO-Twin began in 1991 (for some in 1993) and ended April 2002. These studies were selected because the survey instruments used for each study were similar (Gold et al., 2002) and provided the greatest coverage across the second half of the lifespan in the STR. Furthermore, each study included an identical loneliness item that could be harmonized across studies without altering the response scale. The analytic sample consisted of 2,476 individual twins from 1,632 unique families. This project was approved by the Institutional Review Board at the University of Southern California (ID: UP-17-00067).

SATSA was a community-based longitudinal study of aging that included all same-sex twins who previously indicated to the STR that they were separated prior to 11 years of age and a sample of twins reared together who were demographically matched based on their gender, date, and county of birth (Finkel and Pedersen, 2004). In the current study, loneliness was measured up to six times in 1987, 1990, 1993, 2004, 2007, and 2010. The base sample included 2,840 twins, of whom 1,104 twins provided at least one loneliness score and so were included in the current study.

GENDER was a community-based longitudinal study of opposite-sex twins that focused on sex differences in aging outcomes (Gold et al., 2002). All still-living opposite-sex twins born between 1906 and 1925 were invited to participate, which consisted of 3,398 individual twins. Loneliness was measured up to five times in 1992, 1995, 1999, 2003, and 2007. In the current study, 831 were included because they provided at least one loneliness score.

OCTO-Twin was a community-based longitudinal study of cognitive functioning of the very old (McClearn et al., 1997). Still-living pairs of twins who were at least 80 years of age and had sufficient functional ability to participate in 1.5 h of cognitive testing were invited to participate. Loneliness was measured up to five times in 1991, 1993, 1995, 1997, and 1999. The base sample consisted of 702 individual twins, of which 541 twins were included in the current study because they provided at least one loneliness score.

### 2.2. Measures

#### 2.2.1. Dementia Assessment

Although all OCTO-Twin participants were administered cognitive testing at each wave, only a subset of twins in SATSA and GENDER twins were administered cognitive testing longitudinally (Gold et al., 2002; Finkel and Pedersen, 2004). Participants from all three studies, however, were cognitively screened at least once in the Screening Across the Lifespan Twin Study (SALT; Gatz et al., 2005). Twins who performed poorly or who demonstrated decline from the previous in-person assessment were given an in-person battery that included physical and neuropsychological assessment, blood panels, and depression screening. Clinical diagnoses were made by a diagnostic consensus board using in-person performance and medical records according to DSM-III-R or DSM-IV criteria for dementia and NINCDS-ADRDA criteria.

Twins who were not administered cognitive testing or were lost to follow-up by study design or participant refusal at a later wave were followed by registry linkage, using individual-level data from the Swedish National Patient Register (NPR) and Cause of Death Register (CDR). Registries contained International Classification of Disease (ICD) codes for dementia diagnoses and were made through November 30, 2019. Registry diagnoses were used to supplement clinical diagnoses as long as the diagnoses were made prior to the end of each study: April 1, 2002 for OCTO-Twin; January 1, 2015 for SATSA; and July 1, 2007 for GENDER. In the current study, 586 individual twins were diagnosed with dementia, with 61.09% diagnosed by consensus panel following cognitive and medical assessment and 38.91% diagnosed via one of the Swedish national registries.

Phenotypic and Cox regression analyses used the dichotomous dementia diagnosis variable. For the biometric twin models, Pearson residuals were taken from a logistic regression model in which clinical gold standard was regressed on cohort, sex, study, age at first loneliness assessment, education level, International Socio-Economic Index of occupational status (ISEI), and cumulative illness rating score. We also included an APOE ε4 indicator (0 = no ε4 alleles; 1 = one ε4 allele; 2 = two ε4 alleles). Of the 926 individuals with an APOE genotype (37.40% of the total sample), 0.76% were 2/2 (*n* = 7), 13.93% were 2/3 (*n* = 129), 3.24% were 2/4 (*n* = 30), 56.91% were 3/3 (*n* = 527), 23.11% were 3/4 (*n* = 214), and 2.05% were 4/4 (*n* = 19). Based on prior research including participants with APOE 2/4 (De Luca et al., 2016), we assigned twins with this genotype as having one ε4 allele. Twins' residual scores were used as a proxy of dementia risk because categorical outcomes in multigroup three-level mixed-effects models in M*plus* currently is not possible (Muthén and Muthén, 2017). Residual variance in generalized linear models is not identically distributed. Pearson residuals, thus, were used, as they are comparable to standardized residuals in standard linear regression models (Faraway, 2006). While the distribution of the residuals is non-normal (see Supplementary Figure 1), they are nearly perfectly correlated with clinical diagnosis (*r* = 0.99).

#### 2.2.2. Loneliness

Loneliness was measured with a single item in each study. In OCTO-Twin, the item read, “Presently, does it happen that you get troubled by feelings of loneliness?” In SATSA, the item read, “Are you ever troubled by feelings of loneliness?” In GENDER, the item read, “Are you bothered with feelings of loneliness?” All three items consisted of the same response options: Nearly always/Almost always/Always (1), Often (2), Seldom (3), Hardly ever/Almost never/Never (4). Responses were reverse scored so that higher scores indicate higher loneliness.

### 2.3. Data Analysis

We begin by reporting the sampling characteristics of the total sample. Individual study sampling characteristics are presented in Supplementary Tables 1–3. Cox regression modeling results, in which dementia diagnosis is regressed on loneliness, age at baseline, and the interaction between loneliness and age at baseline, are reported next. We then present the results of a three-level multilevel model to test whether increases in loneliness predicted dementia risk, adjusting for genetic and environmental selection processes hypothesized to confound their association. The Supplementary Material presents a full description of the multilevel model estimation. Linear and non-linear growth curve models were estimated first to test inter-individual differences in intra-individual change in loneliness scores from age 40.40 to age 101.00 are compared. We subsequently decomposed within- and between-family variability in the intercept and slopes into within- and between-family genetic and environmental influences, an approach equivalent to more traditional SEM approaches to quantitative genetic modeling (McArdle and Prescott, 2005). For each random variable—denoted π_0*ij*_ for the intercept, denoted π_1*ij*_ for the linear slope, and denoted π_2*ij*_ for the quadratic slope—the variance is decomposed into within-family and between-family genetic and environmental variance components. The respective expressions for the intercept and slopes are:


(1) $$\begin{array}{l} \pi_{0ij}=A_{w_{0ij}}+E_{w_{0ij}}+A_{b_{00j}}+E_{b_{00j}} \end{array}$$

(2) $$\begin{array}{l} \pi_{1ij}=A_{w_{1ij}}+E_{w_{1ij}}+A_{b_{10j}}+E_{b_{10j}} \end{array}$$

(3) $$\begin{array}{l} \pi_{2ij}=A_{w_{2ij}}+E_{w_{2ij}}+A_{b_{20j}}+E_{b_{20j}} \end{array}$$

At the within-family level, genetic variance components—*A*_*w*_0*ij*__ for the intercept, *A*_*w*_1*ij*__ for the linear slope, and *A*_*w*_2*ij*__ for the quadratic slope—are genetic factors that account for differences in twins' growth parameters of loneliness. Non-shared environmental variance components—*E*_*w*_0*ij*__ for the intercept, *E*_*w*_1*ij*__ for the linear slope, and *E*_*w*_2*ij*__ for the quadratic slope—are environmental factors that account for differences in twins' growth parameters of loneliness. As the growth parameters are modeled at levels 2 and 3, and therefore do not contain measurement error, non-shared environmental variances are unbiased by measurement error. At the between-family level, genetic variance components—*A*_*b*_00*j*__ for the intercept, *A*_*b*_10*j*__ for the linear slope, and *A*_*b*_20*j*__ for the quadratic slope—are genetic factors that account for similarity in twins' growth parameters of loneliness. Shared environmental variance components—*E*_*b*_00*j*__ for the intercept, *E*_*b*_10*j*__ for the linear slope, and for *E*_*b*_20*j*__ the quadratic slope—are any non-genetic factor that accounts for similarity in twins' growth parameters of loneliness.

The within- and between-family genetic effects constitute the total genetic effect, *A*_*ij*_:


(4) $$\begin{array}{l} A_{ij}=A_{w_{ij}}+A_{b_{j}} \end{array}$$

The weights of the within-family and between-family components must be defined ensure that the following conventional twin modeling assumption is met: MZ twins share 100% of their genotype and DZ twins share 50% of their genotype, on average. In the MZ twins, the within-family genetic effect is 0 while the between-family genetic effect has a weight of 1:


(5) $$\begin{array}{l} A_{ij}=0A_{w_{ij}}+1A_{b_{j}} \end{array}$$

In the DZ twins, the total genetic variance is divided equally across the between- and within-levels:


(6) $$\begin{array}{l} A_{ij}=\sqrt{.5}A_{w_{ij}}+\sqrt{.5}A_{b_{j}} \end{array}$$

Estimation of the genetic and environmental variance components is a necessary step in testing the extent to which the genetic and environmental sources of variance underlying individual growth parameters of loneliness predict dementia risk. The dementia residuals, *DemResid*_*ij*_, for twin *i* in family *j* subsequently were regressed on the genetic and environmental components:


(7) $$\begin{array}{l} DemResid_{ij}=\mu +areg_{1}A_{0ij}+areg_{2}A_{1ij}+areg_{3}A_{2ij}\\ \;+creg_{1}E_{b_{0ij}}+creg_{2}E_{b_{1ij}}+creg_{3}E_{b_{2ij}}\\ \;+ereg_{1}E_{w_{0ij}}+ereg_{2}E_{w_{1ij}}+ereg_{3}E_{w_{2ij}}+e_{ij} \end{array}$$

In Equation 7, *areg*_1_, *creg*_1_, and *ereg*_1_ represent the genetic and environmental effects of the intercept on dementia risk; *areg*_2_, *creg*_2_, and *ereg*_2_ represent the genetic and environmental effects of the linear slope on dementia risk; and *areg*_3_, *creg*_3_, and *ereg*_3_ represent the genetic and environmental effects of the quadratic slope on dementia risk. Statistically significant *ereg*_1_, *ereg*_2_, and *ereg*_3_ coefficients support the hypothesis that loneliness predicts dementia risk, statistically adjusting for any genetic and shared environmental correlations between them. Social selection hypotheses may also be supported if there are effects of genetic and/or shared environmental influences of loneliness on dementia risk. Both social selection and social causation hypotheses may be supported simultaneously. The alpha (α) cutoff value was set at 0.05, such that probability values under this value are regarded as statistically significant.

All multilevel models were fit in M*plus* 8.5 (Muthén and Muthén, 2017) using maximum likelihood estimation with robust standard errors (MLR) to account for the non-normal distribution of the dementia residuals. Model fit comparisons were conducted using likelihood ratio tests (LRT), Akaike information criterion (AIC), and Bayesian information criterion (BIC). Less complex models (i.e., models with fewer parameters) were preferred if LRT values were non-significant. Lower AIC and BIC values are preferred, as lower values indicate better relative quality of the model given the data (Burnham and Anderson, 2004).

Missing data were not missing completely at random, as non-response was correlated with the number of loneliness measures contributed to the analysis [*t*_(2,474)_ = 57.05, *p* < 0.001]; time in the study [*t*_(2,474)_ = 15.64, *p* < 0.001]; and sex [$\chi_{\left(1,\text{N}\;=\;2,476\right)}^{2}$ = 5.72, *p* = 0.017]. As missing data in longitudinal studies of aging are plausibly missing not at random (MNAR), direct maximum likelihood estimates that assume data are missing at random (MAR) still may be biased (Enders, 2011). Per current missing data recommendations for multilevel data, multiple imputation was used to handle non-response (Enders et al., 2016). Fifty data sets were imputed using predictive mean matching in the *mice* package in R (van Buuren and Groothuis-Oudshoorn, 2011). The following correlates of missingness were included: time in study, zygosity, sex, cohort, education, APOE ε4 status, and cumulative illness rating scale scores. Data were imputed within the GENDER, OCTO-Twin, and SATSA samples. Within each study, sensitivity analyses were performed to check the severity of violations of the MAR assumption using the δ-adjustment procedure (Van Buuren, 2018). Results suggest that the MAR assumption appeared to be tenable for OCTO-Twin and GENDER, but less so for SATSA. Sensitivity analysis results are reported in the Supplementary Material. Imputed data were then reanalyzed in R (Cox regressions) and M*plus* (multilevel models). Pooled estimates and standard errors are reported.

## 3. Results

### 3.1. Descriptive Results

**Figure 1:**
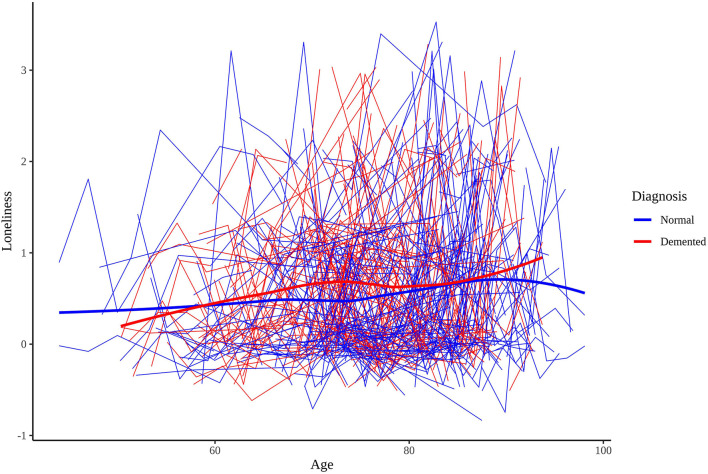
Longitudinal trajectory plots of loneliness scores for 250 cognitively normal and 250 dementia diagnosed participants.

Table 1 presents the sample characteristics. The mean age of the sample at baseline measurement of loneliness 74.75 (*SD* = 8.66), and 25.59% of the sample became demented during the study window. The mean age of dementia onset was 82.67 years (*SD* = 6.29). Participants' mean baseline loneliness scores were 0.60 (*SD* = 0.77) while participants' mean last loneliness scores were 0.68 (*SD* = 0.80). The difference between mean baseline and mean last scores was statistically significant [*t*_(2,475)_ = 4.81, *p* < 0.001], suggesting that loneliness scores tend to increase over time, on average. Both demented and cognitively normal twins showed a tendency to increase in loneliness, on average. Figure 1 presents the longitudinal trajectory plots of loneliness scores for a subset of twins who became demented during the study window (*n* = 250) and a subset of twins who did not (*n* = 250). Demented twins' scores were slightly greater than non-demented twins' scores overall.

**Table 1:** Descriptive statistics of GOS sample.

**Variable**	**Mean (%)**	***SD***	***n***
Dementia	0.24	-	2476
Loneliness (Baseline)	0.60	0.77	2476
Loneliness (Last Q)	0.68	0.80	2476
Age at intake	73.75	8.66	2476
Mean years until diagnosis	9.91	7.51	586
APOE ε4			
0 alleles	74.84	-	693
1 allele	23.11	-	214
2 alleles	2.05	-	19

*Percentages (%) are given for dementia and APOE ε4*.

**Figure 2:**
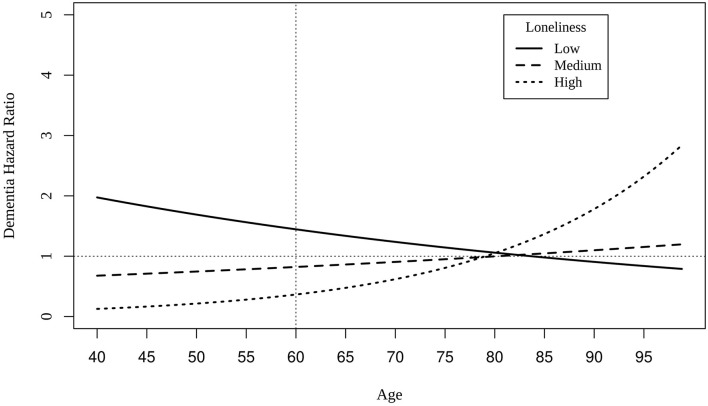
Hazard ratios of dementia for different levels of loneliness as a function of age at intake. The dotted vertical line indicates the centering age of 60, and the dotted horizontal represents hazard ratio value of 1.00. Low = loneliness score of 1; medium = loneliness score of 2; high = loneliness score of 3 (maximum).

Cox regression models indicate that mildly elevated levels of loneliness at baseline (i.e., scores of 1) at the centered intake age of 60 predicted greater risk of dementia (HR: 1.43, 0.95CI: 1.33, 1.52), statistically adjusting for effects of age and the interaction between baseline loneliness score and age. All model results are presented in Supplementary Table 4. Age at intake significantly moderated each level of loneliness. As intake age increased, dementia risk declined for those who reported mildly elevated levels of loneliness, whereas dementia risk increased among those with the highest levels of loneliness, especially after age 80 (Figure 2).

**Figure 3:**
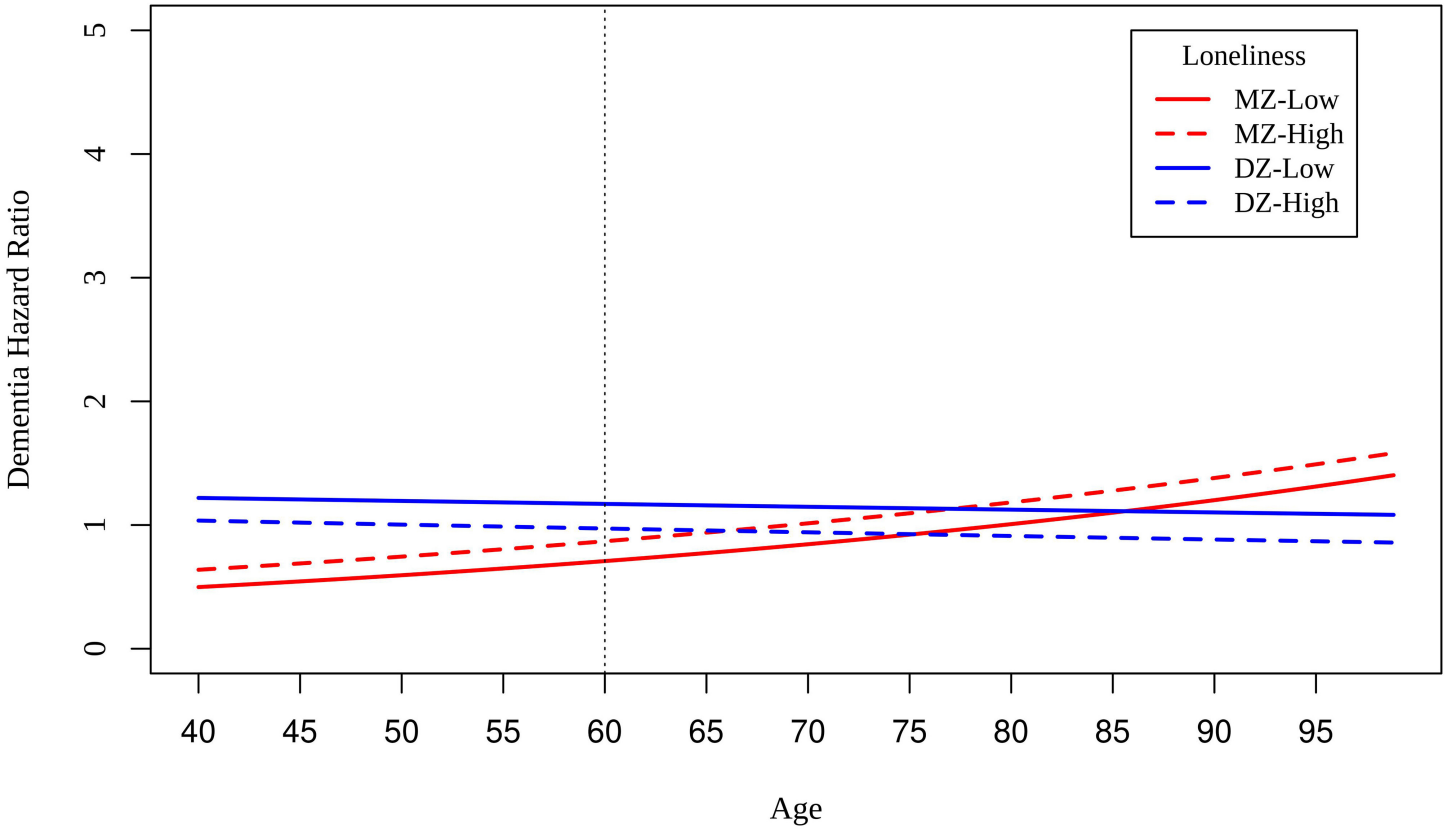
Hazard ratios of dementia for MZ twins (red lines) and DZ twins (blue lines) who are low (solid lines) and high (dashed lines) on loneliness scores at baseline.

We reran Cox regression models in MZ and DZ twins who reported low and high loneliness scores at baseline. Slightly smaller differences in risk between MZ twins compared to DZ twins at the centered intake age of 60 suggests shared genetic variance and shared environmental variance underlying the association between loneliness and dementia risk (Figure 3). Differences in dementia risk are relatively constant across intake age among DZ twins, whereas dementia risk increases with increasing age among MZ twins. Unexpectedly, DZ twins reporting lower loneliness than their co-twins had greater risk of dementia at age of intake.

### 3.2. Multilevel Model Results

Multilevel modeling began by estimating individual growth parameters—intercept, linear slope, and quadratic slope—of loneliness scores as a function of age. Model fit suggested that a quadratic model fit the data best (see Supplementary Table 2), although there was no between-pair variation in the quadratic slope. The shape of the average population trajectory suggests that the curvature of loneliness is convex with respect to age and swings up more dramatically as age deviates further from the centering age of 60 (see Supplementary Figure 1).

Within twins, the phenotypic correlations between the individual growth parameters and dementia risk indicate a negligible positive correlation between the intercept and dementia risk (*r* = 0.01, SE = 0.07), an essentially nil correlation for the linear slope (*r* = 0.0004, *SE* = 0.33), and a negligible correlation for the quadratic slope (*r* = −0.04, *SE* = 0.58). None were statistically significant.

The intraclass correlation coefficients (ICC) in Table 2 indicate that MZ twins are more similar than DZ twins, on average, for all growth parameters, suggesting that genetic variance underlies each random variable. As there was no between-pair variance in the quadratic slope, ICC values could not be estimated. MZ twins were not twice as correlated as DZ twins for the intercept at age 60, suggesting that shared environmental factors also might explain part of the variance in the intercept. The MZ twin correlation for the linear slope is more than twice the DZ twin correlation, as indicated by the negative *c*^2^ value; only genetic factors account for variance in the linear slope. Non-shared environmental sources of variance accounted for the majority of variance in all variables.

**Table 2:** MZ and DZ variance components, intraclass correlations, and univariate genetic and environmental estimates for loneliness growth parameters and dementia risk.

**Random variable**	**MZ_W_**	**MZ_B_**	**DZ_W_**	**DZ_B_**	**ICC_MZ_**	**ICC_DZ_**	***h* ^2^**	***c* ^2^**	***e* ^2^**
Intercept_L_	0.18	0.07	0.15	0.05	0.29	0.25	0.08	0.21	0.71
Linear slope_L_	0.04	0.002	0.007	0.001	0.33	0.14	0.38	−0.05	0.67
Dementia risk	1.03	0.86	1.00	0.76	0.45	0.43	0.04	0.41	0.55

Finally, biometric regression models were fit to the data to test whether genetic and environmental variance components confounded effects of the intercept and linear slope on dementia risk. We tested a model that constrained the genetic and shared environmental variance and regression parameters to be equal to each other found that they could not be equated (likelihood ratio = 14.20, Δ*df* = 2, *p* < 0.001). Yet, none of the regression effects were statistically significant (Table 3), suggesting that effects of the intercept observed above were no longer statistically significant after adjusting for genetic and shared environmental confounds.

**Table 3:** Maximum likelihood estimates from the biometric regression model.

**Parameter**	**Estimate**	***SE***	***p***
A_I_ → Dem	−7.98	77.54	0.918
A_S_ → Dem	−1.94	98.31	0.984
C_I_ → Dem	3.20	3.54	0.904
C_S_ → Dem	-	-	-
E_I_ → Dem	−0.23	1.04	0.825
E_S_ → Dem	3.39	8.68	0.696
$\sigma_{A_{I}}^{2}$	0.02	0.04	0.560
$\sigma_{A_{S}}^{2}$	0.001	0.004	0.870
$\sigma_{C_{I}}^{2}$	0.04	0.03	0.242
$\sigma_{C_{S}}^{2}$	-	-	-
$\sigma_{E_{I}}^{2}$	0.15	0.04	<0.001
$\sigma_{E_{S}}^{2}$	0.01	0.01	0.502
$\sigma_{{Dem}_{MZW}}^{2}$	1.15	4.16	0.782
$\sigma_{{Dem}_{MZB}}^{2}$	0.11	0.07	0.139
$\sigma_{{Dem}_{DZW}}^{2}$	0.55	0.47	0.242
$\sigma_{{Dem}_{DZB}}^{2}$	0.00	0.00	0.999
$\sigma_{age}^{2}$	0.36	0.01	<0.001
σ_*A*_*I*_, *A*_*S*__	0.002	0.003	0.620
σ_*C*_*I*_, *C*_*S*__	-	-	-
σ_*E*_*I*_, *E*_*S*__	0.01	0.01	0.676

*A, additive genetic effect; C, shared environmental effect; E, non-shared environmental effect; Dem, residualized dementia score; I, intercept; S, linear slope; MZ, monozygotic; DZ, dizygotic; W, within-family level; B, between-family level*.

## 4. Discussion

The current study is among the first to evaluate whether individual differences in intraindividual change in loneliness predict dementia risk. Additionally, we leveraged the genetically informative nature of the sample to test whether their correlation is attributed to social selection, social causation, or both. Although we initially observed that elevated baseline levels of loneliness predicted dementia risk, no effect of change in loneliness on dementia was observed. Indeed, the baseline effect of loneliness on dementia risk also was not observed after adjusting for genetic and environmental confounds. Additionally, overall null findings in the biometric regression model suggest support for neither the social causation nor social selection hypotheses. Replication in other longitudinal twin studies is needed to identify the plausible mechanisms that explain how loneliness correlates with dementia risk.

Cox regression model results suggest that mild levels of loneliness increased risk of dementia 1.43 times greater at age 60 than those without loneliness. Although higher levels of loneliness were found to predict decreased risk of dementia at intake age of 60, at intake ages greater than 80, those with the highest levels of loneliness were predicted to have greater risk of dementia compared to those with no loneliness or mild levels (as shown in Figure 2). All effects of loneliness on dementia risk were small, as has been observed in prior research (He et al., 2000; Holmén et al., 2000; Rawtaer et al., 2017; Zhou et al., 2018; Elovainio et al., 2020; Shibata et al., 2020; Sundström et al., 2020; Sutin et al., 2020). Such small overall correlations may explain the null set of findings in the biometric regression models. When small variances are divided among genetic and environmental confounds as well as non-shared environmental effects, significant and meaningful effects surely will be more difficult to detect.

Change in loneliness did not correlate with dementia risk, which is consistent with two recent findings (Akhter-Khan et al., 2021; Lee et al., 2021). In the Framingham Heart Study, for example, only those who reported persistent (i.e., constant) loneliness had increased risk of dementia; participants whose loneliness scores changed across the two waves had decreased or equivalent dementia risk compared to those who reported not feeling lonely at either wave (Akhter-Khan et al., 2021). The average population trajectory in the current study suggests that loneliness is expected to change, with most experiencing worsening levels of loneliness. One possibility is that any change in loneliness, even for worse, may require people to adapt to new emotional states. Resolving and accepting loneliness may require a level of cognitive processing that could protect against dementia. As an example, older adults who experience an uptick in loneliness may take up a new hobby that requires developing a new skill, like painting, that could protect against dementia risk. Conversely, older adults with even a mild level of chronic, constant loneliness may be inured to their lifestyle and seek no compensatory activities to protect against the deleterious effects of loneliness. Null effects also may be an artifact of the sampling procedure, as participants who respond to questionnaires into old-old age must meet a minimum threshold of cognitive functioning regardless of their level of loneliness.

Social selection may have a role in the association between loneliness and dementia risk; current findings could not rule them in or out. Although genetic and shared environmental confounds were non-significant, inclusion of them in the model eliminated the expected effect of the intercept on dementia risk. As both loneliness (Distel et al., 2010; Abdellaoui et al., 2019) and dementia (Beam et al., 2020) are moderately to highly heritable, we were surprised that a genetic correlation between them was not observed. Future twin studies of loneliness and dementia should continue to clarify whether genetic variance partly explains their association.

Several limitations should be considered when interpreting the current findings. First, the number of identified cases of dementia may have been underestimated. The Swedish national registries have a sensitivity of about 50%, which means that a considerable number of twins declared “cognitively normal” may have actually been diagnosed with dementia (Rizzuto et al., 2018). Second, missing responses in longitudinal studies of aging often result from attrition due to death and illness, which means that the missing data mechanism is likely MNAR. Although MNAR mechanisms are difficult to discern and cannot be ruled out definitively (Enders, 2011), we implemented a δ-adjustment procedure (Van Buuren, 2018) that suggests the current results likely held despite departures from the MAR assumption. Third, loneliness was measured with one item. Multivariate measurement of loneliness would provide a more robust construct that may yield different results. Fourth, the bimodal distribution of the dementia residuals means that the standard errors of the biometric regression model should be interpreted cautiously. Finally, twin studies, too, have limitations that could affect the estimation of genetic and shared environmental variance. Conventional twin models assume non-assortative mating by parents, which ensures that DZ twins share half of their genotype, on average. This assumption often is false, which can lead to underestimation of genetic variance.

Current findings add to the growing body of literature on loneliness and dementia risk. We replicated prior findings by showing that mild levels of baseline loneliness predict a 43% increased risk of dementia. Consistent with newly reported findings (Akhter-Khan et al., 2021), change in loneliness did not correlate significantly with dementia risk. Future research should investigate whether chronicity of loneliness is a key feature that increases risk of dementia.

## Data Availability Statement

The data analyzed in this study is subject to the following licenses/restrictions: The data include variables protected by the Karolinska Institute in Sweden. Requests to access these datasets should be directed to https://ki.se/en/research/swedish-twin-registry-for-researchers.

## Ethics Statement

The studies involving human participants were reviewed and approved by University of Southern California Institutional Review Board. The patients/participants provided their written informed consent to participate in this study.

## Author Contributions

CB: conception of the work and acquisition of data. CB, AK, AG, and ML: analysis and interpretation of data for the work. AJK, AG, LF, MP, ML, CC, EP, AK, and CB: writing and revising the work and final approval of the version to be published and agreement to be accountable for all aspects of the work. All authors contributed to the article and approved the submitted version.

## Funding

This work was supported by the National Institutes of Health, National Institute on Aging (T32 AG000037, R01 AG060470, and RF1 AG058068) and the Alzheimer's Association (AARF-17-505302).

## Conflict of Interest

The authors declare that the research was conducted in the absence of any commercial or financial relationships that could be construed as a potential conflict of interest.

## Publisher's Note

All claims expressed in this article are solely those of the authors and do not necessarily represent those of their affiliated organizations, or those of the publisher, the editors and the reviewers. Any product that may be evaluated in this article, or claim that may be made by its manufacturer, is not guaranteed or endorsed by the publisher.
