# Supplementary material for: A Genetically Informed Longitudinal Study of Loneliness and Dementia Risk in Older Adults
Source: Front Genet. 2021 Sep 17;12:661474. doi: 10.3389/fgene.2021.661474 (PMC8484886; doi:10.3389/fgene.2021.661474)
Supplement: Supplementary file 1 [file Data_Sheet_1.pdf]

Supplementary Material

1 SUPPLEMENTARY METHODS

Multilevel model fitting began by fitting three growth curve models: an unconditional means model (Model 1), an unconditional linear growth model (Model 2), and an unconditional quadratic growth model (Model 3). The following describes the most complex model, the unconditional quadratic growth model. Level 1 models the within-person change in loneliness and is specified as:

$$L_{ijt} = \pi_{0ij} + \pi_{1ij} Age_{ijt} + \pi_{2ij} Age^2_{ijt} + \epsilon_{ijt} \tag{S1}$$

Equation S1 expresses loneliness scores, *L*, for person *i* in family *j* over *t* measurements of loneliness. Age was centered at 60 years. The parameter π_{0ij} is the random intercept at age 60; π_{1ij} is the random linear slope that represents the instantaneous rate of change at age 60; and π_{2ij} is the random quadratic slope that represents the curvature of the growth trajectory or the change in the rate of change (i.e., swing) in loneliness as twins deviate from age 60. Finally, ϵ_{ijt} represents the residual loneliness scores, which are assumed to be independent and identically distributed over persons and age. The random intercept and slopes consist of a mean and variance component, as all twins have their own intercept and slope terms. This is represented in the following set of level 2 expressions:

$$\pi_{0ij} = \beta_{00j} + r_{0ij} \tag{S2}$$

$$\pi_{1ij} = \beta_{10j} + r_{1ij} \tag{S3}$$

$$\pi_{2ij} = \beta_{20j} + r_{2ij} \tag{S4}$$

In equation S2, the random intercept, π_{0ij}, is a function of β_{00j}, which is the mean within-family intercept across all twins and a deviation score, r_{0ij}, which is the variance of the random intercept within each family. In equation S3, the random linear slope, π_{1ij}, is a function of β_{10j}, which is the mean within-family linear slope across all twins and a deviation score, r_{1ij}, which is the variance of the random linear slope. In equation S4, the random quadratic slope, π_{2ij}, is a function of β_{20j}, which is the mean within-family quadratic slope across all twins and a deviation score, r_{2ij}, which is the variance of the random quadratic slope. Because twins are nested within-families, variance in level 2 intercepts and slopes can further be decomposed into between-family variance components at level 3:

$$\beta_{00j} = \gamma_{000} + u_{00j} \tag{S5}$$

$$\beta_{10j} = \gamma_{100} + u_{10j} \tag{S6}$$

$$\beta_{20j} = \gamma_{200} + u_{20j} \tag{S7}$$

In equation S5, β_{00j} is a function of the grand mean of the random intercept, γ_{000}, and a between-family variance component, u_{00j}, of the intercept. In equation S6, β_{10j} is a function of the grand mean of the random linear slope, γ_{100}, a between-family variance component, u_{10j}, of the linear slope. In equation S7, β_{20j} is a function of the grand mean of the random quadratic slope, γ_{200}, and a between-family variance component, u_{20j}, of the quadratic slope.

We fit three models to identify the general form of the longitudinal trajectory of loneliness. The baseline model was a random intercept-only model that models individual differences in constant levels of loneliness over age. The second model was an unconstrained linear growth model that includes both a random intercept and random linear slope term. The third model was an unconstrained quadratic growth model that includes a random intercept, a random linear slope, and a random quadratic slope. All models were fit in M*plus* 8.4 (Muthén & Muthén, 1998-2017) using full information maximum likelihood estimation. Missing data were assumed to be missing at random given the use of maximum likelihood. Model fit comparisons were conducted using likelihood ratio tests (LRT), Akaike Information Criterion (AIC), and Bayesian Information Criterion (BIC).

2 SUPPLEMENTARY RESULTS

2.1 Multilevel Models

The initial steps of the multilevel modeling comprised of fitting an unconditional means model, unconditional linear growth model, and unconditional quadratic growth model. Model fit and comparisons are provided in Table S5. In the unconditional quadratic growth model, the intercept, linear slope, and quadratic slope means and variances were statistically significant. This model fit the data significantly better than the intercept-only model (Model 1) and the unconditional linear growth model (Model 2). The mean intercept was 0.50 (*SE* = 0.03, $p < .001$) with a person-level variance of 0.14 (*SE* = 0.03, $p < .001$). The large variance suggests notable individual differences in participants' predicted loneliness scores at age 60. The mean linear slope was 0.02 (*SE* = 0.03, $p < .385$) with a person-level variance of 0.01 (*SE* = 0.03, $p < .694$). Because the unconditional quadratic slope model fit the data best, the linear slope is not constant across the age window, but represents the instantaneous slope at age 60. The mean quadratic slope was 0.04 (*SE* = 0.01, $p < .001$) with a person-level variance of 0.002 (*SE* = 0.01, p = .798).

2.2 MNAR Sensitivity Analysis

The δ-adjustment sensitivity approach was used to evaluate whether severity of violations of the MAR assumption led to biased parameter estimates. For this analysis, we estimated the hazard ratios of each level of loneliness in Model 1 of the Cox regressions presented in Table S4. The δ-adjustment ranged from 0 (no violation of MAR) to 1.00 (severe violation of MAR). Under this approach, a fixed amount (i.e., 0, -.25, -.50, -.75, or -1.00) is subtracted from the imputed values. Cox regressions were re-run for each adjustment to the imputed values, and hazard ratios are compared across the different δ values. The MAR assumption of the multiple imputation procedure can be trusted to the extent that adjustments do not influence parameter estimates. Results are presented in Tables S6-S8. Hazard ratios in OCTO-Twin and GENDER did not vary as much across the different δ-adjustments as did estimates in SATSA. Overall, values were relatively stable, which suggests that the MAR assumption mostly held in these data. Still, MNAR mechanisms cannot be tested directly, and it must be noted that these adjustments simulate only four different MNAR scenarios among the infinite MNAR mechanisms that could affect the data.

3 SUPPLEMENTARY TABLES AND FIGURES

Table S1. Descriptive statistics of SATSA sample

Variable	Mean (%)	*SD*	*n*
Dementia	0.27	-	1104
Loneliness (Baseline)	0.68	0.76	1104
Loneliness (Last Q)	0.78	0.79	1104
Age at intake	68.78	9.10	1104
Mean years until diagnosis	14.13	7.77	302
APOE ϵ4			
0 alleles	70.65	-	207
1 allele	26.96	-	79
2 alleles	2.39	-	7

Note. Percentages (%) are given for dementia and APOE ϵ4.

Table S2. Descriptive statistics of GENDER sample

Variable	Mean (%)	*SD*	*n*
Dementia	0.20	-	831
Loneliness (Baseline)	0.54	0.74	831
Loneliness (Last Q)	0.55	0.73	831
Age at intake	74.11	3.86	831
Mean years until diagnosis	6.72	3.82	163
APOE ϵ4			
0 alleles	75.21	-	270
1 allele	22.28	-	80
2 alleles	2.51	-	9

Note. Percentages (%) are given for dementia and APOE ϵ4.

Table S3. Descriptive statistics of OCTO-Twin sample

Variable	Mean (%)	*SD*	*n*
Dementia	0.22	-	541
Loneliness (Baseline)	0.54	0.85	541
Loneliness (Last Q)	0.65	0.89	541
Age at intake	83.32	3.01	541
Mean years until diagnosis	3.70	2.51	121
APOE ϵ4			
0 alleles	78.83	-	216
1 allele	20.07	-	55
2 alleles	1.09	-	3

Note. Percentages (%) are given for dementia and APOE ϵ4.

Table S4. Cox regression model results

	Model 1		Model 2		Model 3	
Variable	HR	95% CI	HR	95% CI	HR	95% CI
Loneliness (1)	**1.24**	**1.22–1.26**	**1.17**	**1.15–1.19**	**1.43**	**1.33–1.52**
Loneliness (2)	**0.94**	**0.90–0.98**	0.96	0.92–1.00	0.83	0.68–1.01
Loneliness (3)	0.90	0.78–1.05	1.00	0.86–1.16	0.38	0.08–1.94
Age	-	-	**0.9514**	**0.9513–0.9514**	**0.9543**	**0.9542–.9544**
Age:Loneliness (1)	-	-	-	-	**0.9845**	**0.9843–0.9848**
Age:Loneliness (2)	-	-	-	-	**1.01**	**1.009–1.0104**
Age:Loneliness (3)	-	-	-	-	**1.054**	**1.05–1.058**

Note. Bolded values are statistically significant (p $<$ .05). Log likelihood (LL): Model 1 = -4381.39 (df = 3); Model 2 = -4337.41 (df = 4); Model 3 = -4334.74 (df = 7). Model 2 compared to Model 1: Likelihood ratio (LR) = 87.95 (Δdf = 1), $p < .001$. Model 3 compared to Model 2: LR = 5.36 (Δdf = 3), p = .148.

Table S5. Growth curve model fit results

Model	-LL	Parameters	LR	p	AIC	BIC
Intercept Only	-14460.19	4	-	-	28928.39	28958.42
Linear Model	-14260.75	8	398.88	<.001	28537.50	28597.58
Quadratic Model	-14255.56	12	55.48	.034	28535.11	28625.22

Note. The unconstrained linear growth model is compared to the intercept only model, and the unconstrained quadratic growth model is compared to the unconstrained linear growth model. -LL = negative log-likelihood; LRT = likelihood ratio; AIC = Akaike Information Criterion; BIC = Bayesian Information Criterion.

Table S6. MNAR Sensitivity Analysis for OCTO-Twin

	δ-adjustment				
Parameter	0	-.25	-.50	-.75	1.00
Loneliness (1)	0.94	0.93	0.93	0.94	0.93
Loneliness (2)	0.96	0.96	0.96	0.97	0.97
Loneliness (3)	1.65	1.62	1.64	1.63	1.63

Note. Reported values are hazard ratios.

Table S7. MNAR Sensitivity Analysis for SATSA

	δ-adjustment				
Parameter	0	-.25	-.50	-.75	1.00
Loneliness (1)	1.10	1.13	1.13	1.10	1.12
Loneliness (2)	0.90	0.87	0.97	0.93	0.96
Loneliness (3)	0.87	0.95	0.96	0.88	0.94

Note. Reported values are hazard ratios.

Table S8. MNAR Sensitivity Analysis for GENDER

	δ-adjustment				
Parameter	0	-.25	-.50	-.75	1.00
Loneliness (1)	1.24	1.23	1.24	1.23	1.23
Loneliness (2)	0.91	0.91	0.92	0.91	0.91
Loneliness (3)	0.25	0.25	0.25	0.25	0.25

Note. Reported values are hazard ratios.

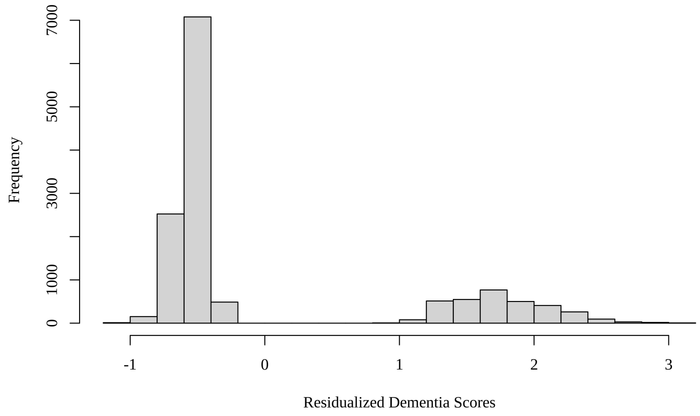

Figure S1. Histogram of the Pearson residuals of dementia diagnosis. Pearson residuals were estimated from a logistic regression model in which diagnosis was regressed on cohort, sex, and study.

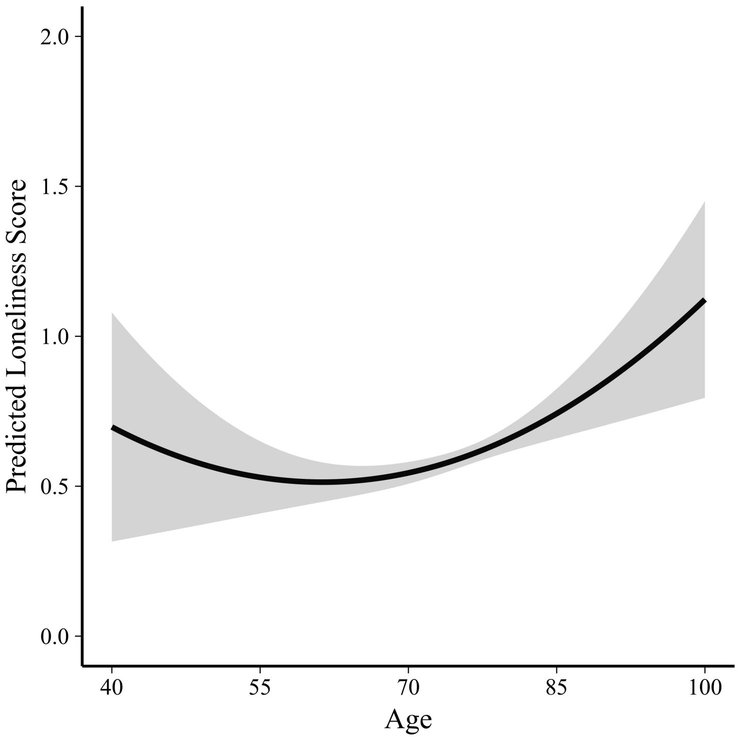

Figure S2. Model estimated longitudinal trajectory of loneliness. Maximum likelihood estimates and standard errors from the raw data were used to describe the shape and confidence bands.
